# The Physical Symptom Scale-8: Psychometric Characteristics of a Short-Form Version of the PHQ-15 and its Use in TMD-Related Assessment and Research

**DOI:** 10.11607/ofph.3187

**Published:** 2023-11-17

**Authors:** Adrian Ujin Yap, Darren Zong Ru Lee, Sharon Hui Xuan Tan

**Affiliations:** Department of Dentistry; Ng Teng Fong General Hospital and Faculty of Dentistry; National University Health System, Singapore;; National Dental Research Institute Singapore, National Dental Centre Singapore, and Duke-NUS Medical School, Singapore Health Services;; School of Health and Social Sciences; School of Health and Social Sciences; Saw See Hock School of Public Health; National University of Singapore, Singapore;; School of Health and Social Sciences

**Keywords:** pain measurement, reliability and validity, reproducibility of results, somatic symptoms, temporomandibular joint disorders

## Abstract

**Aims:**

To describe the development of the Physical Symptom Scale-8 (PSS-8) and to examine its psychometric properties and use in temporomandibular disorder (TMD)–related assessment and research.

**Methods:**

An online survey comprising demographic variables, the DC/TMD pain screener (TPS), Short-Form Fonseca Anamnestic Index (SFAI), PSS-8, PHQ-15, and Depression, Anxiety, and Stress Scale-21 (DASS-21) was administered to young adults attending a technical college. The PSS-8 adopted the Somatic Symptom Scale-8 (SSS-8) items but maintained the 3-point response scale and 4-week time frame of the PHQ-15. Internal consistency and reliability of the PSS-8 were determined by its Cronbach α value. Known-groups and concurrent/convergent validity were examined using Mann-Whitney *U* test and Spearman correlation (α = .05), respectively.

**Results:**

Responses from 400 participants (mean age 18.8 ± 1.5 years; 52.3% women) were evaluated. Pain-related (WPT) and all (WAT) TMDs were present in 8.5% and 17.3% of the sample, respectively. The PSS-8 exhibited good internal consistency (α = 0.82) and sound known-groups validity, with the WPT/WAT groups having significantly higher PSS-8 scores than those without TMDs. Good concurrent and convergent validity were also observed, with moderate to strong correlations with the PHQ-15 (*rs* = 0.97) and DASS-21 scores (*rs* = 0.48 to 0.60). Correlations with the TPS and SFAI scores were weaker (*rs* = 0.28 to 0.34).

**Conclusion:**

The PSS-8 presented good psychometric properties and performed similarly to the PHQ-15. It holds promise as the “de facto” shortened version of the PHQ-15 for TMDs and related work. *J Oral Facial Pain Headache 2023;37:159–165. doi: 10.11607/ofph.3187*

Temporomandibular disorders (TMDs) are a diverse group of musculoskeletal conditions distinguished by pain and/or dysfunction of the temporomandibular joints (TMJs), masticatory muscles, and adjoining structures.^1^ They present a significant public health problem, with prevalence rates of up to 16% and 75% when established via formal diagnostic criteria and self-reported surveys/physical examinations, respectively.^2,3^ TMDs are about two times more common in women, who constitute 80% of TMD patients.^4^ The etiology of TMDs is multifaceted and congruous with the biopsychosocial model of illness.^5^ Psychologic distress, including depression, anxiety, and stress, is often associated with TMDs.^6,7^ Studies have indicated that up to 60% of TMD patients experience moderate to severe depression and 77% suffer moderate to severe somatization.^8^ Given the latter, some have considered TMDs to be a type of functional somatic syndrome (FSS).^9^ This is supported by the high comorbidity between TMDs and other FSS, such as chronic fatigue syndrome and fibromyalgia, found in previous literature.^10,11^

Somatization is the tendency to “experience and communicate” psychologic distress in the form of physical (somatic) symptoms.^12^ Asian populations appear to be more susceptible to somatization and have higher levels of somatic symptoms, including bodily pains and dizziness, than Western populations.^13,14^ This phenomenon has been explained by the overt emphasis on somatic “idioms” of distress and stigma accompanying mental health problems in Asian cultures.^15^ More recently, TMDs and somatic symptoms were posited as manifestations of psychologic distress and predicted by female sex, anxiety, and stress in Asian youths.^16^ These “idioms” or cultural concepts of distress must be integrated into health assessments, interventions, and research to personalize care, improve study validity, and facilitate equitable data comparison across multicultural settings.^17^

The Patient Health Questionnaire-15 (PHQ-15) is a reliable, valid, and pragmatic measure for assessing the severity of physical symptoms and somatization.^18–20^ Furthermore, it is equivalent or superior to other concise inventories for examining physical symptoms and screening for somatoform disorders (a group of psychiatric conditions causing medically unexplained somatic symptoms).^20,21^ The PHQ-15 is applicable in diverse health care situations and was incorporated into Axis II of the Diagnostic Criteria for TMDs (DC/TMD) standard for appraising nonspecific physical symptoms.^22^ Despite its good psychometric properties and popularity, the PHQ-15 contains several items that may not be relevant to all people. Moreover, shorter questionnaires are desirable, as they could increase participation rates and reduce item nonresponse, response fatigue, and administration time.^23,24^ The Somatic Symptom Scale-8 (SSS-8) is an abbreviated 8-item version of the PHQ-15^25^ developed as a brief patient-reported measure of physical symptom burden for the Diagnostic and Statistical Manual of Mental Disorders fifth edition (DSM-5) field trials.^26^ Three PHQ-15 items on fainting, sexual, and menstrual problems were omitted because of their low prevalence and weak associations with other items, such as quality of life, functionality, and health care use measures. Additionally, 5 PHQ-15 items concerning cardiovascular and gastrointestinal symptoms were condensed into 2 items, S1 and S5, which are presented in Table 1. However, a 5-point response scale and a 1-week time frame (as opposed to the 3-point scale and 4-week time frame of the PHQ-15) were assumed to emulate the response options offered for other measures in the DSM-5 field trials.^26^ Considering the chronic and recurrent nature of TMDs/FSS, which often extends beyond a week, and to mirror the 4-week time frame employed by the Symptom Questionnaire (SQ) of the DC/TMD,^9,22^ it is rational that the SSS-8 be revised to follow the original structure of the PHQ-15.

**Table 1:** Items of the PSS-8 and Their Applicability

During the past 4 weeks, how much have you been bothered by any of the following problems?				
Item no.		Not bothered (0 points)	Bothered a little (1 point)	Bothered a lot (2 points)
S1	Stomach or bowel problems	180 (45.0)	194 (48.5)	26 (6.5)
S2	Back pain	161 (40.3)	178 (44.5)	61 (15.3)
S3	Pain in the arms, legs, or joints	180 (45.0)	177 (44.3)	43 (10.8)
S4	Headaches	177 (44.3)	173 (43.3)	50 (12.5)
S5	Chest pain or shortness of breath	249 (62.3)	122 (30.5)	29 (7.3)
S6	Dizziness	238 (59.5)	131 (32.8)	31 (7.8)
S7	Feeling tired or having low energy	117 (29.3)	176 (44.0)	107 (26.8)
S8	Trouble sleeping	183 (45.8)	148 (37.0)	69 (17.3)

Data are reported as n (%).

The aims of this project were thus to develop the PSS-8, which applies the items of the SSS-8 but maintains the 3-point response scale and 4-week time frame of the PHQ-15, and to establish its psychometric characteristics and use in TMD-related assessment and research. The research hypotheses were that the PSS-8 *(1)* has high internal consistency and reliability, *(2)* has good known-groups, concurrent, and convergent validity, and *(3)* is useful for TMD-related work.

## Materials and Methods

### Study Design and Sample

This project is part of a survey on the prevalence of somatic, TMD, and psychologic symptoms in Asian young adults (institutional review board approval number: SHS2018-005). The minimum sample size of 204 participants was computed based on a 95% confidence level, a CI of ± 5%, a student population of 14,700, and a 16% prevalence rate of TMDs with an online sample size calculator (https://www.calculator.net).^2^ Study subjects were enlisted from a technical college that trains diploma students for the local workforce using a stratified (by gender) random sampling technique. Except for a history of orofacial trauma or surgical procedures in the previous 2 weeks, there were no other exclusion criteria. No remunerations were offered for contributing to the study. All participants provided informed consent before answering an online survey encompassing demographic variables, the TMD pain screener (TPS) of the DC/TMD, Short-Form Fonseca Anamnestic Index (SFAI), PSS-8, PHQ-15, and Depression, Anxiety, and Stress Scales-21 (DASS-21).^18,27–29^

### Study Measures

The presence of painful (PT) TMDs and all TMDs (AT), comprising PT and/or intra-articular (IT) TMDs, was established with the TPS and SFAI. Both TMD screening inventories have high sensitivity (99% for TPS; up to 98% for SFAI) and specificity (97% for TPS; up to 97% for SFAI).^27,30^ Furthermore, the SFAI presents high diagnostic accuracy for detecting DC/TMD–defined PT, IT, and AT, with area under the receiver operating characteristic curves (AUCs) of 0.99, 0.97, and 0.97, respectively.^30^ The TPS consists of 6 items relating to jaw/temple pain patterns, jaw pain/stiffness when first awake, and aggravating/relieving activities in the last 30 days (4-week time frame). Response options are specified with “a” = 0 points, “b” = 1 point, and “c” = 2 points. A total TPS score exceeding the cutoff of 3 points indicates that PTs are present. The SFAI was developed to address the multidimensionality of the 10-item FAI and enhance its accuracy.^28,31^ The SFAI contains 5 items on TMD pain (arthralgia and myalgia) and intra-articular TMJ symptoms (TMJ sounds, opening, and side movement difficulties). Items are scored on a 3-point frequency scale with “no” = 0 points, “sometimes” = 5 points, and “yes” = 10 points. A total SFAI score ≥ 15 indicates the presence of ATs. For both TPS and FAI, higher scores signify more/greater severity of TMD symptoms. The participants were subsequently dichotomized into those without PT/AT (NPT/NAT) and with PT/AT (WPT/WAT) for statistical analyses.

The PSS-8 and PHQ-15 involve 8 and 15 nonspecific physical symptoms, respectively. Table 1 displays the items of the PSS-8 that were adopted from the SSS-8. Both the PSS-8 and PHQ-15 are assessed over a 4-week time frame and scored on a 3-point applicability scale varying from “not bothered at all” (0 points) to “bothered a lot” (2 points). Total PSS-8 scores range from 0 to 16 points, while total PHQ-15 scores span from 0 to 30 points. For the latter, total scores of 5, 10, and 15 points serve as the cutoff points for mild, moderate, and severe physical symptoms, respectively. Negative affectivity (NA) and emotional states were examined with the DASS-21. The measurement properties of the DASS-21 are well established and have been reviewed systematically.^32^ This instrument involves 21 items with 7 questions dedicated to the subscales of depression, anxiety, and stress. The items are scored on a 4-point applicability scale varying from “did not apply to me at all” (0 points) to “applied to me very much, or most of the time” (3 points). Total DASS and subscale scores are calculated to ascertain the gravity of NA; that is, the propensity for experiencing negative emotional states, as well as depressive, anxiety, and stress symptoms.^33^ Greater scores suggest higher levels of NA/emotional distress and cutoff points for the various severity groupings (normal to extremely severe), which are obtainable from the DASS manual.^29^

### Statistical Analyses

Statistical evaluations were conducted using Stata Statistical Software Release 14, with the significance level set at .05. While categoric data were stated as frequencies with proportions, numeric data were conveyed as means with SDs and medians with interquartile ranges. The internal consistency reliability of the PSS-8 was determined by computing its Cronbach α. Alpha values from 0.6 to 0.7 and > 0.8 indicate satisfactory and good reliability, respectively.^34^ The internal consistency of the PSS-8 was further examined via the serial elimination of individual items. Any increase in α coefficients implies that the item does not relate to the others, and a corrected item-rest correlation of ≥ 0.20 was deemed adequate. Nonparametric statistical methods were applied, as the data were not normally distributed based on Shapiro-Wilk test. Known-groups validity was explored using Mann-Whitney *U* test, and concurrent/convergent validity was confirmed by correlating PSS-8 scores with the PHQ-15, TPS, SFAI, and DASS-21 scores using Spearman ρ correlation. Correlation coefficients (*rs*) were subsequently categorized as weak, moderate, or strong based on cut-off values of 0.1, 0.4, and 0.7, respectively.^35^

## Results

### Study Sample

Data from a total of 400 participants were evaluated (response rate of 63.6%). Table 2 shows the demographics and distribution of the study sample, whose mean age was 18.8 ± 1.5 years and comprised 52.3% women. PTs and ATs were present in 8.5% and 17.3% of the cohort, respectively. Although differences in age and sex distributions were not statistically significant, the proportion of women with PT (WPT = 67.7%) and AT (WAT = 59.4%) were observed to be higher. Regarding somatic symptoms, the participants were most bothered by feeling tired/having low energy (26.8%), trouble sleeping (17.3%), back pain (15.3%), and headaches (12.5%) based on the PSS-8 (Table 1).

**Table 2:** Demographics and Distribution of the Study Sample (N = 400)

Variables	Total sample	Pain-related TMDs			All TMDs		
		NPT	WPT	*P*	NAT	WAT	*P*
n (%)	400	366 (91.5)	34 (8.5)		331 (82.8)	69 (17.3)	
**Age, y**							
Mean ± SD	18.8 ± 1.5	18.7 ± 1.5	19.3 ± 1.9	.099	18.7 ± 1.5	19.0 ± 1.7	.198^a^
Median (IQR)	19 (1)	19 (1)	19 (2)		19 (1)	19 (2)	
**Gender**							
Women, n (%)	209 (52.3)	186 (50.8)	23 (67.7)	.060	168 (50.8)	41 (59.4)	.190^b^
Men, n (%)	191 (47.8)	180 (49.2)	11 (32.4)		163 (49.2)	28 (40.6)	

^a^Mann-Whitney *U* test .

^b^Chi-square test (*P* < .05).

### Reliability

The Cronbach α values of the PSS-8 and PHQ-15 (all items) were 0.82 and 0.86, respectively. Table 3 presents the internal consistency of the various PSS-8 items. Even with serial omission of individual items, the α values remained high and close to 0.8. Corrected item-rest correlation coefficients ranged from 0.47 to 0.62 and were well above the 0.2 thresholds. The weakest and strongest item-rest correlations were noted for S1 (stomach/bowel problems) and S4 (headaches), respectively.

**Table 3:** Internal Consistency and Reliability of the PSS-8 Items

PSS-8	Cronbach α if item excluded (n = 400)	Corrected item-rest correlation
S1	0.81	0.47
S2	0.80	0.55
S3	0.81	0.50
S4	0.79	0.62
S5	0.80	0.55
S6	0.80	0.61
S7	0.81	0.53
S8	0.81	0.54

### Validity

The mean/median PSS-8, PHQ-15, and DASS-21 scores for various TMD groupings are displayed in Table 4. The WPT and WAT groups were observed to have significantly greater PSS-8 and PHQ-15 scores than the NPT and NAT groups (*P* < .001). Furthermore, they also reported significantly higher DASS total, depression, anxiety, and stress scores than their counterparts with no TMDs (*P* < .01). Correlation coefficients between the various variables are reflected in Table 5. The PSS-8 was strongly correlated with the PHQ-15 (*rs* = 0.97). Moreover, correlations with the DASS total, depression, anxiety, and stress scores were moderately strong (*rs* = 0.48 to 0.60), with NA/anxiety and stress exhibiting the highest correlations. Though significant, the associations of PSS-8 with TPS and SFAI scores were relatively weaker (*rs* = 0.28 to 0.34). Despite being considerably shorter, the PSS-8 and PHQ-15 had comparable validity.

**Table 4:** Mean/Median PSS-8, PHQ-15, and DASS-21 Scores for Various Groups

Variables	Pain-related TMDs			All TMDs		
	NPT	WPT	*P*	NAT	WAT	*P*
**PSS-8**						
Mean ± SD	5.11 ± 3.49	7.68 ± 4.32	< .001	4.85 ± 3.45	7.64 ± 3.62	< .001
Median (IQR)	5 (6)	7.5 (8)		5 (5)	7 (6)	
**PHQ-15**						
Mean ± SD	7.04 ± 5.13	11.6 ± 6.65	< .001	6.66 ± 5.06	11.09 ± 5.64	< .001
Median (IQR)	6 (7)	12.5 (11)		6 (7)	10 (8)	
**DASS-21**						
Total						
Mean ± SD	26.07 ± 23.12	46.82 ± 33.10	< .001	24.27 ± 22.29	44.96 ± 28.75	< .001
Median (IQR)	20 (30)	36 (42)		18 (30)	40 (40)	
**Depression**						
Mean (SD)	8.95 ± 9.31	14.94 ± 12.94	.008	8.44 ± 9.25	14.35 ± 10.92	< .001
Median (IQR)	6 (12)	11 (20)		6 (14)	14 (18)	
**Anxiety**						
Mean ± SD	7.56 ± 7.04	14.82 ± 9.74	< .001	7.05 ± 6.70	13.62 ± 9.06	< .001
Median (IQR)	6 (10)	12 (14)		6 (8)	14 (12)	
**Stress**						
Mean ± SD	9.56 ± 8.75	17.06 ± 11.87	< .001	8.79 ± 8.34	16.99 ± 10.56	< .001
Median (IQR)	8 (14)	14 (16)		6 (12)	16 (14)	

Mann-Whitney *U* test (*P* < .05).

**Table 5:** Correlation Coefficients Between Variables

Variables	PSS-8	PHQ-15	TPS	SFAI	DASS-21: Total	DASS-21: Depression	DASS-21: Anxiety
**PSS-8**	–	–	–	–	–	–	–
**PHQ-15**	0.97**	–	–	–	–	–	–
**TPS**	0.28**	0.30**	–	–	–	–	–
**SFAI**	0.34**	0.37**	0.41**	–	–	–	–
**DASS-21 Total**	0.60**	0.61**	0.30**	0.39**	–	–	–
**DASS-21 Depression**	0.48**	0.48**	0.24**	0.30**	0.92**	–	–
**DASS-21 Anxiety**	0.57**	0.58**	0.30**	0.41**	0.89**	0.72**	–
**DASS-21 Stress**	0.60**	0.61**	0.29**	0.38**	0.94**	0.79**	0.79**

Spearman correlation. **P < .001.

## Discussion

In the present study, a short-form version of the PHQ-15 (PSS-8) was created, and the psychometric properties of the abbreviated measure were evaluated. As the PSS-8 had good reliability and validity, and individuals with and without TMDs can be distinguished by their PSS-8 scores, the research hypotheses were all supported. Based on the TPS and SFAI, pain-related and all TMDs were present in 8.5% and 17.3% of the study cohort, respectively. The prevalence rates of painful and all TMDs were consistent with those reported for the general population, specifically 9.7% for myalgia, 11.4% for disc displacements, and 2.6% for arthralgia.^2^ The TPS/SFAI and PHQ-15 have been used jointly with the DASS-21 in other studies.^16,36,37^ The DASS-21 is the sole psychologic measure that assesses depressive, anxiety, and stress symptoms simultaneously. While the stress subscale considers tension, nervous arousals, agitation, and impatience, the anxiety subscale gauges situational anxiety, bodily arousals/symptoms, and anxious effects. The depression subscale quantifies the state of hopelessness, low mood, and self-esteem.^29^ A general factor of NA, which is described by the DASS-21 total score, has been found to contribute to all three subscales.^33,38^ Moreover, NA in addition to stress, preceding life events, and somatic symptoms also predicted the onset of TMDs.^39^

### Reliability

Both the PSS-8 and PHQ-15 showed good internal consistency and reliability, with α values of > 0.8. Such high α values were also observed for other language versions of the PHQ-15 (α = 0.81 to 0.83) involving very large study samples.^40,41^ Alpha values stayed around 0.8 despite the sequential exclusion of individual items, attesting to the robust interconnections among the different PSS-8 items. Although headaches were the third most troublesome physical symptom, they had the strongest association with all other somatic complaints. These findings support those of Tietjen et al, who reported a high prevalence of severe physical symptoms in persons with chronic/disabling headaches and proposed a “psychobiologic” basis for the synergistic relationships between depression, physical symptoms, and headaches.^42^

### Validity

Known-groups validity was established, with the WPT/WAT groups posing significantly higher PSS-8 scores than the NPT/NAT groups. Individuals with PTs and ATs could hence be set apart by their physical symptom scores. Following the PHQ-15 severity categorization, preliminary cutoff points for mild, medium, and high physical symptoms for the PSS-8 might be 3, 5, and 8 points after adjustment for the 8 items. The WPT and WAT groups therefore experienced moderate physical symptom burden based on both the PSS-8 and PHQ-15. The association between TMDs and somatization/physical symptoms has been deliberated and is probably mediated by psychosocial distress.^8,9,39^ The PSS-8 showed very good concurrent validity when compared to the PHQ-15 (*rs* = 0.97). Toussaint et al compared the SSS-8 to the PHQ-15 (minus the item on sexual problems) and observed a good, albeit weaker, association (*rs* = 0.79).^43^ The PSS-8, with its 4-week time frame and 3-point response scale, may therefore better parallel the PHQ-15. These findings also suggest that the discounted items of the PHQ-15 might not be relevant or prevalent in young adults with TMDs.

Good convergent validity was confirmed, with moderately strong correlations between the PSS-8 and NA/DASS-21 subscales (*rs* = 0.48 to 0.60). The NA, anxiety, and stress subscales presented the strongest associations with the PSS-8 (*rs* ≈ 0.60). Psychologic stress has been found to trigger “exaggerated and blunted” sympatho-adrenal-medullary (SAM) and hypothalamic-pituitary-adrenal (HPA) system responses that predict future mental and physical health/illness outcomes, including depression, anxiety, and musculoskeletal pain.^44^ Correlation coefficients were akin to those of the PHQ-15 (*rs* = 0.48 to 0.61) as well as those of the SSS-8 to other instruments measuring anxiety and depression (*rs* = 0.37 to 0.53).^43^ The associations between the PSS-8 and TPS/SFAI scores were relatively weaker *(rs* = 0.28/0.34); likewise between the PHQ-15 and TPS/SFAI scores (rs = 0.30/0.37). This could be explained partly by the involvement of nonclinical community young adults as opposed to clinical TMD patients in this study. Nonetheless, outcomes were consistent with the weak correlations between the Brief Pain Inventory, which measures pain severity and impact on daily functioning, and the SSS-8/PHQ-15 (*rs* = 0.36/0.28) in veterans suffering from pain and comorbid depression/anxiety.^43^

### Study Limitations

The present study has several limitations. First, the study involved only young adults, representing the peak incidence age for TMDs.^1^ The PSS-8 also needs to be investigated in older adults as well as in medical patients to confirm its appropriateness for the general population. Second, the response rate, though adequate (> 60%) for health surveys, may still be subjected to some nonresponse bias.^45^ Then again, research participation rates have fallen over the last decade (increase in nonparticipation/nonresponse) due to the decrease in social contributions, increase in life complexities, involvement liabilities (including time requirements), and privacy law changes.^46^ Third, as the study measures were all self-administered, outcomes may be disposed to self-reporting, social desirability, recall, confirmation, and other partialities.^47^ Finally, only the English version of the PSS-8 was evaluated. The modified scale must be translated into other languages and psychometrically tested before it can be universally adopted.

## Conclusions

In the present study, the PSS-8 was created and its psychometric properties, as well as utility, characterized in a cohort of young adults with and without TMDs. The PSS-8 showed good internal consistency (Cronbach α = 0.82) and sound known-groups validity, with the WPT/WAT groups displaying significantly greater PSS-8 scores when compared to those without TMDs. Good concurrent and convergent validity were also noted, with strong and moderately strong correlations with the PHQ-15 (*rs* = 0.97) and DASS-21 scores (*rs* = 0.48 to 0.60), respectively. Correlations with the TPS and SFAI scores were weaker (*rs* = 0.28 to 0.34). Despite being considerably shorter, the PSS-8 presented equivalent outcomes to the PHQ-15 and holds promise as the “de facto” short-form version of the PHQ-15 for TMDs and other related work.

## Key Findings

- The PSS-8 presented good internal consistency (Cronbach α = 0.82) and sound known-groups validity.
- The PSS-8 exhibited good concurrent validity, with a strong correlation with the PHQ-15 (*rs* = 0.97), and good convergent validity, with moderately strong correlations with the DASS-21 (*rs* = 0.48 to 0.60).
- The PSS-8 could serve as the short-form version of the PHQ-15.
